# Risk factors for surgical site infection requiring reoperation after osteotomy around the knee: a multicenter retrospective study

**DOI:** 10.1186/s12891-026-10277-2

**Published:** 2026-07-23

**Authors:** Yuji Arai, Shuji Nakagawa, Yusei Katsuyama, Atsuo Inoue, Koichi Idei, Shohei Ichimaru, Keisuke Sugie, Kentaro Hayashi, Kenji Takahashi

**Affiliations:** 1 Department of Sports and Para-Sports Medicine, Graduate School of Medical Science, Kyoto Prefectural University of Medicine, 465 Kajiicho, Kawaramachi-Hirokoji, Kamigyo-ku, Kyoto, 602-8566 Japan; 2 Department of Orthopaedics, Graduate School of Medical Science, Kyoto Prefectural University of Medicine, Kyoto, 602-8566 Japan; 3 Department of Orthopedics, Kanai Hospital, Kyoto, 613-0911 Japan; 4 Department of Orthopedics, Koseikai Takeda Hospital, Kyoto, 600-8558 Japan

**Keywords:** Osteotomy around the knee, Surgical site infection, Risk factors, Retrospective study

## Abstract

**Background:**

Osteotomy around the knee (OAK) is an established joint-preserving procedure for knee osteoarthritis; however, postoperative surgical site infection (SSI) remains a serious complication that may compromise bone healing and functional outcomes. Evidence regarding risk factors for SSI requiring reoperation after OAK is limited. This study aimed to identify perioperative factors associated with SSI requiring reoperation following OAK.

**Methods:**

This multicenter retrospective study included 247 patients (302 knees) who underwent OAK at one institution and two affiliated hospitals between June 2015 and June 2024. SSI was defined as an infection requiring surgical debridement. Patient demographics, comorbidities, perioperative laboratory data, and surgical variables were evaluated. Factors associated with SSI were identified using Firth’s penalized multivariable logistic regression. Sensitivity analyses were performed after excluding knees that underwent osteotomy for valgus deformity and after restricting the dataset to one knee per patient.

**Results:**

SSI requiring reoperation occurred in 12 of 302 knees (4.0%). Firth’s penalized multivariable logistic regression identified preoperative skin disease, preoperative liver disease, and postoperative hemoglobin decline as factors independently associated with SSI. These associations remained consistent in sensitivity analyses excluding knees that underwent osteotomy for valgus deformity and restricting the analysis to one knee per patient.

**Conclusions:**

Preoperative skin disease, preoperative liver disease, and postoperative hemoglobin decline were independently associated with an increased risk of SSI requiring reoperation after OAK. Awareness of these factors may aid perioperative risk assessment and postoperative surveillance. Further prospective studies with larger cohorts are needed to validate these findings and establish clinically applicable strategies for infection risk assessment and prevention.

## Background

Osteotomy around the knee (OAK) is an established joint-preserving surgical option for patients with knee osteoarthritis, particularly in relatively young and active individuals. Through correcting lower limb alignment and redistributing mechanical load across the knee joint, OAK can relieve pain, improve function, and delay the need for total knee arthroplasty [1–7].

Despite these favorable outcomes, postoperative complications remain a clinical concern [8–10]. Surgical site infection (SSI) requiring reoperation is a serious complication as it can compromise bone healing, necessitate implant removal, prolong hospitalization, and adversely affect functional outcomes. Reported infection rates following OAK vary widely, reflecting differences in patient populations, surgical techniques, and definitions of infection [11].

Several risk factors for postoperative infection have been identified in other orthopedic procedures, including patient-related factors such as comorbidities and perioperative anemia, as well as surgery-related factors such as operative time and blood loss. However, evidence specifically addressing risk factors for SSI after OAK remains limited. Moreover, OAK has unique characteristics—including extensive soft-tissue dissection, implantation of fixation devices, and dependence on bone healing—that may influence susceptibility to postoperative infection differently from arthroplasty or fracture surgery.

Identifying patients at increased risk of SSI after OAK may contribute to improved perioperative management and postoperative surveillance. However, given the relatively low incidence of infection, robust data to guide risk stratification are lacking, and predictive models have not been well established.

Therefore, the purpose of this multicenter retrospective study was to identify perioperative factors associated with SSI requiring reoperation following OAK.

## Methods

### Study design and ethics approval

This retrospective multicenter study was approved by the Ethics Committee of the Kyoto Prefectural University of Medicine (ERB-C-2046).

### Participants

Consecutive patients who underwent OAK for meniscal injury, knee osteoarthritis, idiopathic knee osteonecrosis, or varus deformity at one institution and two affiliated hospitals between June 2015 and June 2024 were retrospectively analyzed. Indications for OAK included failure of conservative treatment for more than three months, predominantly involving medialor lateral compartment pathology, with preserved range of motion. No restrictions were imposed regarding sex, body weight, age, osteoarthritis grade, degree of lower-limb deformity, or anterior cruciate ligament status.

**Fig. 1:**
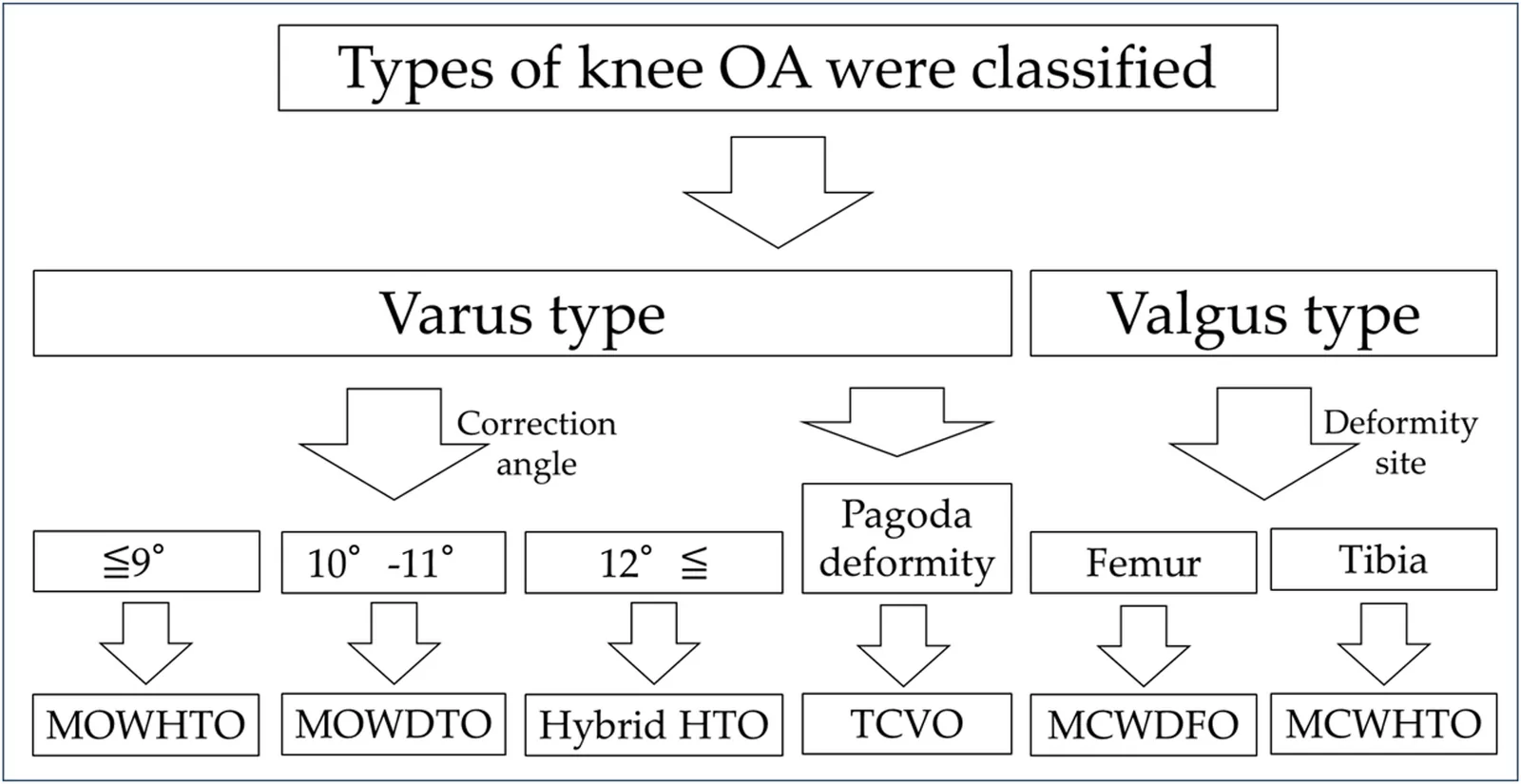
Flowchart of OAK procedure selection. Abbreviations: OA: osteoarthritis; MOWHTO: medial open wedge osteotomy; MOWDTO: medial open wedge distal tuberosity osteotomy; TCVO: tibial condylar valgus osteotomy; MCWDFO: medial closed wedge distal femoral osteotomy; MCWHTO: medial closed wedge high tibial osteotomy

Patients were excluded if they demonstrated advanced degeneration in non-target compartments, symptoms attributable to these compartments, flexion contracture ≥ 10°, or low activity levels. Knee osteoarthritis was classified as varus-type or valgus-type, and the surgical procedure was selected according to a predefined institutional algorithm (Fig. 1). In varus-type osteoarthritis cases with a postoperative medial proximal tibial angle ≥ 95°, double-level osteotomy was selected and excluded from the present analysis.

All procedures were performed by six orthopedic surgeons, each with more than 10 years of experience, using standardized surgical indications and techniques. Inclusion criteria were osteotomy of the proximal tibia or distal femur, fixation with a single Tris plate^®^ (Olympus Terumo Biomaterials, Tokyo, Japan), and postoperative follow-up of at least three months. After exclusions, 247 patients (302 knees) were included.

The cohort comprised 93 male patients (114 knees) and 154 female patients (188 knees). Unilateral procedures were performed in 192 patients, with bilateral procedures performed in 55 patients. No patients required perioperative blood transfusion. Intraoperative blood loss was generally minimal, and no cases of massive bleeding or bleeding-related complications were observed. The mean postoperative follow-up period was 37 (range, 3–101) months. Patient characteristics are summarized in Table 1.

**Table 1:** The demographic characteristics of the study population (*n* = 302)

Parameter	Total
Number of knees	302
Number of patients	247
Age (years)	62 (22–84)
Gender (male)	114 (38%)
BMI (kg/m^2^)	24.36 (16.71–49.26)
Smoke	101 (33%)
Alcohol	143 (47%)
Comorbidities	
DM	49 (16%)
RA	11 (4%)
Endocrine disease	14 (5%)
Malignant disease	22 (8%)
Kidney disease	3 (1%)
Liver disease	11 (4%)
Skin disease	5 (2%)
Knee joint diagnoses	
OA	235 (78%)
ON	18 (6%)
Meniscal tear, MM/LM	43 (14%)/2 (1%)
Varus deformity	4 (1%)
Surgical techniques	
MOWHTO	216 (72%)
MOWDTO	15 (5%)
Hybrid HTO	56 (19%)
MCWDFO	10 (3%)
MCWHTO	2 (1%)
TCVO	3 (1%)

Abbreviations: *BMI *body mass index, *DM *diabetes mellitus, *RA *rheumatoid arthritis, *OA *osteoarthritis, *ON *osteonecrosis, *MM *medial meniscus, *LM *lateral meniscus, *MOWHTO *medial open wedge osteotomy, *MOWDTO *medial open wedge distal tuberosity osteotomy, *MCWHTO *medial closed wedge high tibial osteotomy, *TCVO *tibial condylar valgus osteotomy, *MCWDFO *medial closed wedge distal femoral osteotomy

### Surgical technique and postoperative management

For all target alignments, the correction for varus deformity was set at 62.5% of the tibial plateau width (Fujisawa point), whereas for valgus deformity, the target was the top of the medial intercondylar ridge [12–14]. All patients were placed in the supine position under general anesthesia. Arthroscopic examination was performed in all cases, and when meniscal injury was identified, partial meniscectomy or repair was conducted based on the type and severity of the lesion. Medial open-wedge high tibial osteotomy (MOWHTO) was performed using the technique described by Arai et al. [12], and medial open-wedge distal tuberosity osteotomy (MOWDTO) was conducted using the technique described by Akiyama et al. [5]. Skin incisions were made either straight or slightly curved, and all patients received artificial bone grafts. Hybrid HTO was conducted in accordance with Takeuchi et al.’s technique [15]. Medial closing wedge distal femoral osteotomy (MCWDFO) was performed based on Nakamura et al.’s established protocol [6], and tibial condylar valgus osteotomy (TCVO) was performed according to Chiba et al.’s method [16]. In medial closing wedge high tibial osteotomy (MCWHTO), as in MOWHTO, the hinge point was set on the lateral side of the tibia, and a wedge-shaped section on the medial side was removed and closed. Tris plates^®^ (Olympus Terumo Biomaterials, Tokyo, Japan) were used for fixation in all procedures. Postoperative rehabilitation included range of motion exercises starting on the day following surgery, with no limitations based on the surgical technique used. Full weight-bearing was permitted at 3–4 weeks postoperatively for MOWHTO, and at 4–6 weeks for MOWDTO, hybrid HTO, MCWDFO, MCWHTO, and TCVO. Blood tests were conducted preoperatively and at one week postoperatively.

### Definition of surgical site infection

SSI was defined as a postoperative infection requiring surgical reoperation (surgical debridement with or without implant removal), accompanied by clinical signs of infection and elevated inflammatory markers. Therefore, the present definition specifically reflects relatively severe SSI requiring surgical intervention and does not include superficial or conservatively managed infections.

### Data collection

Demographic data, comorbidities, operative variables, and perioperative laboratory values were extracted from the patients’ medical records. Postoperative hemoglobin decline was defined as the difference between the preoperative and one-week postoperative hemoglobin value. Skin disease was defined as dermatologic conditions with active or chronic skin lesions identified preoperatively, including but not limited to asteatotic eczema, stasis dermatitis, nummular eczema, bullous pemphigoid, and neurofibromatosis. Liver disease was defined as preoperatively diagnosed hepatic disorders, including hepatitis B, hepatitis C, and fatty liver disease. Due to the limited number of infection events, these conditions were analyzed as aggregated categories rather than as individual disease entities.

### Statistical analysis

Continuous variables are presented as mean ± standard deviation or median with interquartile range, as appropriate. Categorical variables are presented as counts and percentages. Comparisons between knees with and without SSI were performed using Student’s t-test or Mann–Whitney U test for continuous variables and chi-square test or Fisher’s exact test for categorical variables, as appropriate. Variables with a p-value < 0.10 in univariate analysis were entered into a multivariable logistic regression model to identify factors associated with SSI requiring reoperation. Given the limited number of SSI events and the potential risk of sparse-data bias, the multivariable model was subsequently re-fitted using Firth’s penalized logistic regression [17–19], and these results were considered the primary analysis. Odds ratios (ORs) and 95% confidence intervals (CIs) were calculated. Sensitivity analyses were performed by (1) excluding osteotomy for valgus deformity and (2) restricting the dataset to the first operated knee in patients who underwent bilateral procedures to assess the robustness of the identified associations. Variables with *p* < 0.05 were considered statistically significant. All statistical analyses were performed using EZR (Saitama Medical Center, Jichi Medical University, Saitama, Japan), which is a graphical user interface for R (The R Foundation for Statistical Computing, Vienna, Austria). A two-sided p-value < 0.05 was considered statistically significant.

## Results

### Incidence of SSI

No patients required perioperative blood transfusion, and intraoperative blood loss was generally minimal, with no bleeding-related complications observed. SSI requiring reoperation following OAK occurred in 12 of 302 knees (males, 7 knees; females, 5 knees), corresponding to an overall infection rate of 4%. The median patient age at the time of surgery was 62 (range, 22–84) years, and the median BMI was 24.36 (range, 16.71–49.26) kg/m².

The knee joint diagnoses included 11 knees with osteoarthritis and 1 knee with a medial meniscus posterior root tear. The following patient background factors were identified among the cases with infection: a history of smoking (*n* = 6), alcohol consumption (*n* = 3), diabetes mellitus (*n* = 3), malignancies (*n* = 3), skin diseases (*n* = 2), and liver diseases (*n* = 2).

Preoperative blood test results were as follows (median [range]): albumin, 4.3 (3.3–5.1) g/dL; glomerular filtration rate (GFR), 91.2 (5.0–170.0) mL/min/1.73 m²; preoperative hemoglobin 13.8 (10.5–18) g/dL; in addition to postoperative hemoglobin, 12.1 (8.5–16.4) g/dL and change in hemoglobin (Δhemoglobin) 1.6 (-0.8–4.0) g/dL.

Surgical procedures included MOWHTO (9 cases), MOWDTO (2 cases), and hybrid HTO (1 case). The median operative time was 2 h 4 min (range, 1 h 23 min–3 h 46 min). The median time to infection onset was 107 days postoperatively (range, 16–363 days). The causative organisms identified were methicillin-sensitive *Staphylococcus aureus* (5 knees), methicillin-resistant *Staphylococcus aureus* (1 knee), methicillin-sensitive coagulase-negative *Staphylococcus* (1 knee), *Group B Streptococcus* (1 knee), and unknown (4 knees). The specific treatment strategies and outcomes concerning each case are detailed in Table 2.

**Table 2:** The demographic profile of the population with infection

Gender	age	Skin disease	Liver disease	ΔHb	Surigical technique	Prognostic score	Time to infection (days)	Culture	First reoperation	Second reoperation	Outcome	F/U (m)
M	77	-	-	1.4	MOWHTO	1.4	235	unknown	DAIR	Debridement and plate removal 23 days later	no recurrence	76
M	71	-	-	2.2	hybridHTO	2.2	34	MRSA	DAIR	Debridement and plate removal 156 days later	no recurrence	48
M	64	-	-	2.6	MOWDTO	2.6	46	MSCNS	DAIR and artificial bone removal	N/A	no recurrence plate removal at 678 days	28
M	66	-	-	2.7	MOWHTO	2.7	262	MSSA	Debridement and plate removal	N/A	no recurrence	69
F	67	-	-	1.9	MOWHTO	1.9	49	unknown	DAIR	N/A	no recurrence plate removal at 392 days	62
M	66	-	-	3.3	MOWHTO	3.3	119	MSSA	DAIR	N/A	plate removal at 308 days recurrence	52
F	58	-	HCV	1.4	MOWHTO	4.4	199	unknown	DAIR	Debridement and plate removal at 39 days later	no recurrence	30
M	57	asteatotic eczema	-	3.1	MOWHTO	7.1	268	MSSA	Debridement and plate removal	N/A	no recurrence	46
F	65	-	MASLD	2.5	MOWHTO	5.5	16	unknown	DAIR	N/A	no recurrence plate removal at 357 days	34
M	71	-	-	2.5	MOWDTO	2.5	81	MSSA	DAIR	N/A	no recurrence plate removal at 591 days	35
F	38	-	-	1.7	MOWHTO	1.7	363	GBS	DAIR	N/A	no recurrence plate removal at 427 days	19
F	64	asteatotic eczema	-	1.9	MOWHTO	5.9	95	MSSA	Debridement and plate exchange CLAP	N/A	no recurrence plate removal at 279 days	9

Abbreviation: *M *male, *F *female, *HCV *Hepatitis C, *MASLD *non-alcoholic fatty liver disease, *MOWHTO *medial open wedge osteotomy, *MOWDTO *medial open wedge distal tuberosity osteotomy, *MSSA*: methicillin-susceptible Staphylococcus aureus; MRSA: methicillin-resistant staphylococcus aureus; MSCNS: methicillin-susceptible coagulase-negative staphylococci; GBS: Group B Streptococcus; DAIR: debridement and implant retention

### Risk factor analysis

Following a comprehensive univariate analysis of each factor, conducted separately for 290 knees in the non-infected group and 12 knees in the infected group, no statistically significant differences were observed in patient background variables such as age, sex, body mass index, smoking history, and alcohol consumption. However, notable differences were observed in the prevalence of malignant disease, liver disease, and skin disease. Among the blood test parameters, significant differences were identified in the GFR and Δhemoglobin, whereas no significant differences were observed regarding the osteotomy site or operative time (Table 3).

**Table 3:** The results of the univariate analysis for infection

Parameter	No Infection	Infection	*p* value
	*n* = 290	*n* = 12	
Age (years)	61 (22–84)	65.5 (38–77)	0.253
Gender (male/female)	107/183	7/5	0.138
Smoke	95/195	6/6	0.225
Alcohol	139/151	4/8	0.386
DM	46/244	3/9	0.42
Malignant disease	22/268	3/9	0.067*
Endocrine disease	14/276	0/12	1
RA	11/279	0/12	1
Kidney disease	3/287	0/12	1
Skin disease	3/287	2/10	0.0136*
Liver disease	9/281	2/10	0.0653*
BMI	24.33 (16.71–49.26)	24.61 (20.29–38.47)	0.948
glucose (mg/dL)	100 (73–233)	104 (85–167)	0.683
Alb (g/dL)	4.3 (3.3–5.1)	4.6 (3.9–5.1)	0.157
GFR (ml/min/1.72m2)	91.5 (5.0-170.0)	76.4 (59.5–130.0)	0.0847*
PreHb (g/dL)	13.8 (10.5–18)	14.8 (11.8–16)	0.209
PostHb (g/dL)	12.1 (8.5–16.4)	11.9 (10.1–13.8)	0.832
ΔHb (g/dL)	1.6 (-0.8-4.0)	2.35 (1.4–3.3)	0.00928*
Osteotomy site (femur/tibia)	10/280	0/12	1
Operation time (min)	138.5 (57–292)	124 (83–223)	0.676

Abbreviation: *BMI *body mass index, *DM *diabetes mellitus, *RA *rheumatoid arthritis, *GFR *glomerular filtration rate, *ALB *albumine, *Hb *hemoglobin, *Ag*: argentum, Bold with *: statistically significant

A multivariate logistic regression analysis was subsequently performed, including variables with *p*-values < 0.1 in the univariate analysis—namely, malignant disease, liver disease, skin disease, GFR, and Δhemoglobin—as well as age and sex, which were included as essential patient background factors. The analysis findings showed statistically significant associations for skin disease, liver disease, and Δhemoglobin. To address concerns regarding sparse-data bias, the multivariable analysis was repeated using Firth’s penalized logistic regression. Skin disease, liver disease, and postoperative hemoglobin decline remained significantly associated with SSI (Table 4). A sensitivity analysis was then performed after excluding 12 knees that underwent osteotomy for valgus deformity. The results remained consistent, with skin disease, liver disease, and postoperative hemoglobin decline continuing to show significant associations with SSI (Table 5). To account for potential within-patient clustering, an additional sensitivity analysis was conducted by restricting the dataset to one knee per patient, retaining only the first-operated knee in patients who underwent bilateral procedures. Among the resulting 235 knees, SSI occurred in 9 knees (3.8%). Firth’s penalized logistic regression demonstrated findings that were consistent with the primary analysis, with skin disease, liver disease, and postoperative hemoglobin decline remaining significantly associated with SSI (Table 6).

**Table 4:** Factors Associated with Surgical Site Infection Requiring Reoperation Identified by Firth’s Penalized Logistic Regression Analysis (302 Knees)

Parameter	OR (95% CI)	*p*-value
Age (years)	1.021 (0.964, 1.094)	0.508
ΔHb (g/dL)	2.139 (1.055, 4.739)	0.03420*
Skin disease	31.845 (2.734, 355.541)	0.00784*
Gender	3.813 (0.648, 30.460)	0.144
GFR (ml/min/1.72m^2^)	0.994 (0.957, 1.035)	0.758
Liver disease	12.630 (1.789, 85.737)	0.01360*
Malignant disease	3.084 (0.549, 13.286)	0.181

Abbreviation: *OR *Odds ratio, *CI *Confidence interval, *GFR *glomerular filtration rate, *Hb *hemoglobin, Bold with *: statistically significant

**Table 5:** Sensitivity Analysis Excluding Osteotomies for Valgus Cases: Factors Associated with Surgical Site Infection Requiring Reoperation Identified by Firth’s Penalized Logistic Regression Analysis (290 Knees)

Parameter	OR (95% CI)	*p*-value
Age (years)	1.018 (0.961, 1.092)	0.568
ΔHb (g/dL)	2.151 (1.057, 4.806)	0.03390*
Skin disease	30.947 (2.670, 346.372)	0.00818*
Gender	3.704 (0.633, 29.507)	0.152
GFR (ml/min/1.72m^2^)	0.994 (0.957, 1.035)	0.755
Liver disease	12.300 (1.742, 83.837)	0.01450*
Malignant disease	3.011 (0.536, 12.991)	0.19

Abbreviation: *OR *Odds ratio, *CI *Confidence interval, *GFR *glomerular filtration rate, *Hb *hemoglobin, Bold with *: statistically significant

**Table 6:** Sensitivity Analysis Restricted to One Knee per Patient: Factors Associated with Surgical Site Infection Requiring Reoperation Identified by Firth’s Penalized Logistic Regression Analysis (235 Knees)

Parameter	OR (95% CI)	*p*-value
Age (years)	0.996 (0.937, 1.071)	0.906
ΔHb (g/dL)	3.483 (1.371, 9.917)	0.00841*
Skin disease	62.238 (3.690, 1364.955)	0.00682*
Gender	3.491 (0.431, 50.042)	0.253
GFR (ml/min/1.72m^2^)	0.975 (0.939, 1.019)	0.235
Liver disease	25.993 (2.901, 355.097)	0.00470*
Malignant disease	3.377 (0.523, 18.502)	0.184

Abbreviation: *OR *Odds ratio, *CI *Confidence interval, *GFR *glomerular filtration rate, *Hb *hemoglobin, Bold with *: statistically significant

## Discussion

This multicenter retrospective study identified perioperative factors associated with SSI requiring reoperation after OAK. The principal findings were that preoperative skin disease, preoperative liver disease, and postoperative hemoglobin decline were significantly associated with the occurrence of SSI. These associations remained consistent across multiple sensitivity analyses, supporting the robustness of the findings. Nevertheless, given the limited number of infection events and the retrospective study design, the results should be interpreted with appropriate caution.

In this study, the incidence of SSI requiring reoperation was 4.0%, which is consistent with previously reported infection rates following OAK [10, 11, 20–29]. Furthermore, the reported infection rate of 4% should be interpreted as the incidence of severe SSI requiring reoperation, rather than overall SSI.

Large-scale registry-based and systematic studies have shown that although deep or reoperation-requiring infections post-OAK are relatively uncommon, they remain clinically important owing to their association with prolonged treatment, delayed bone union, implant-related complications, and compromised functional outcomes. The infection rate observed in this cohort therefore aligns with existing literature and supports the clinical relevance of identifying OAK-specific risk factors.

Among the identified factors, preoperative liver disease was shown to be significantly associated with SSI. Chronic liver disease is known to impair host immune function through mechanisms such as monocyte–macrophage dysfunction, altered complement activity, and increased bacterial translocation. In addition, coagulopathy, thrombocytopenia, and hypoalbuminemia commonly associated with liver disease may adversely affect wound healing and increase susceptibility to postoperative infection [30–32]. In the context of OAK, which involves extensive bone cutting, implant fixation, and soft-tissue manipulation, these systemic vulnerabilities may further increase infection risk. These findings underscore the importance of careful preoperative evaluation and optimization of hepatic function when planning OAK.

Preoperative skin disease was also strongly associated with SSI in this cohort. Chronic inflammatory dermatologic conditions, including atopic dermatitis, eczema, and psoriasis, are characterized by impaired skin barrier function and increased colonization by *Staphylococcus aureus* [33]. Previous studies involving other orthopedic procedures, such as anterior cruciate ligament reconstruction and total knee arthroplasty, have reported an increased risk of postoperative infection in patients with these conditions [34, 35]. Given that OAK typically requires relatively long skin incisions and implant placement near the skin surface, compromised skin integrity may play a particularly important role. These findings suggest that preoperative dermatologic optimization and meticulous perioperative skin management may be particularly relevant for patients undergoing OAK.

Postoperative hemoglobin decline was identified as an associated factor for SSI. In the present cohort, no patients required perioperative blood transfusion, indicating that hemoglobin decline primarily reflected perioperative blood loss rather than transfusion-related effects. Rather than representing a direct causal factor, postoperative hemoglobin decline may function as a surrogate marker for perioperative bleeding, hematoma formation, or local tissue hypoxia, all of which may predispose patients to infection. Accordingly, this factor should be interpreted as an indicator for closer postoperative monitoring rather than as a modifiable preoperative risk factor. Tranexamic acid has been shown to reduce perioperative blood loss in other orthopedic procedures; however, its use was not standardized in this study, and the potential effect of antifibrinolytic therapy on SSI risk after OAK warrants further investigation [36]. In addition, although postoperative hemoglobin decline was associated with SSI, this variable was measured after surgery and does not represent a true preoperative risk factor. In the present cohort, hemoglobin values were obtained at one week postoperatively, which preceded the diagnosis of infection in all cases; however, this factor should be interpreted as a postoperative indicator rather than a predictor usable for preoperative risk stratification.

To address concerns regarding sparse-data bias and the limited number of SSI events, additional analyses were performed using Firth’s penalized logistic regression. Furthermore, sensitivity analyses were conducted by excluding osteotomy for valgus deformity and by restricting the dataset to one knee per patient to account for potential within-patient clustering. Across these analyses, the associations of preoperative skin disease, preoperative liver disease, and postoperative hemoglobin decline with SSI remained largely unchanged. These findings support the robustness of the identified associations despite the relatively small number of infection events. Nevertheless, the wide confidence intervals observed for some variables indicate residual statistical uncertainty, and the present findings should be interpreted as exploratory and require validation in larger cohorts.

### Limitations

This study has several limitations. First, it was a retrospective multicenter study, and the number of SSI events requiring reoperation was limited. Although Firth’s penalized logistic regression and multiple sensitivity analyses were performed to reduce sparse-data bias and assess the robustness of the findings, the relatively small number of events resulted in wide confidence intervals for some variables. Therefore, the identified associations should be interpreted as exploratory and hypothesis-generating rather than definitive causal risk factors. In addition, external validation was not performed, and future studies with larger sample sizes are required to confirm the present findings.

Second, the unit of analysis was the knee rather than the individual patient, and some patients contributed bilateral cases. To address potential within-patient clustering, a sensitivity analysis restricted to one knee per patient was performed, and the principal findings remained largely unchanged. Similarly, sensitivity analysis excluding osteotomy for valgus deformity yielded consistent results. Nevertheless, because the cohort predominantly consisted of osteotomies for varus cases, caution is warranted when generalizing these findings to populations with a higher proportion of osteotomy for valgus deformity.

Third, the postoperative follow-up period varied among patients, and some patients were followed for as little as three months. Although all infections requiring reoperation in this cohort were identified during the early postoperative period, late-onset infections after osteotomy have been reported. Therefore, the true incidence of SSI may have been underestimated.

Fourth, perioperative management was not completely standardized across participating institutions. For example, tranexamic acid administration was determined according to institutional or surgeon preference. Such differences may have influenced perioperative blood loss and postoperative hemoglobin decline, and residual confounding cannot be excluded.

Finally, postoperative hemoglobin decline was evaluated as a surrogate marker of perioperative blood loss and physiological stress. However, this parameter may also be influenced by factors such as preoperative anemia, perioperative fluid management, and other unmeasured variables. Therefore, the observed association should not be interpreted as evidence of a direct causal relationship between hemoglobin decline and SSI.

## Conclusions

In this retrospective multicenter study, preoperative skin disease, preoperative liver disease, and postoperative hemoglobin decline were associated with an increased risk of SSI requiring reoperation after OAK. These associations remained consistent across multiple sensitivity analyses and may contribute to perioperative risk awareness and postoperative surveillance in patients undergoing OAK. However, given the limited number of infection events and the retrospective study design, the findings should be considered exploratory and interpreted with caution. Larger prospective studies with adequate event numbers and external validation are required to confirm these associations and to establish clinically applicable strategies for infection prevention and perioperative risk assessment.

## Data Availability

The original contributions presented in this study are included in the article. Further inquiries can be directed to the corresponding author.

## Abbreviations

OAK: Osteotomy Around the Knee
SSI: Surgical Site Infection
CI: Confidence Interval
OR: Odds Ratio
MOWHTO: Medial Open-Wedge High Tibial Osteotomy
MOWDTO: Medial Open-Wedge Distal Tuberosity Osteotomy
MCWDFO: Medial Closing Wedge Distal Femoral Osteotomy
TCVO: Tibial Condylar Valgus Osteotomy
MCWHTO: Medial Closing Wedge High Tibial Osteotomy
GFR: Glomerular Filtration Rate

## References

[1] Ollivier B, Berger P, Depuydt C, Vandenneucker H. Good long-term survival and patient-reported outcomes after high tibial osteotomy for medial compartment osteoarthritis. Knee Surg Sports Traumatol Arthrosc. 2021;29:3569–84. 10.1007/s00167-020-06262-4. Epub 2020 Sep 9. PMID: 32909057. 32909057

[2] Komaki S, Nakagawa S, Arai Y, Inoue A, Kaihara K, Hino M et al. Cartilage degeneration of patellofemoral joint occurs in open wedge high tibial osteotomy, rather than in hybrid closed wedge high tibial osteotomy, during the early postoperative period: A qualitative analysis using MRI T2 mapping. J Orthop Surg (Hong Kong). 2023 Jan-Apr;31:10225536221151132. 10.1177/10225536221151132. PMID: 36757867. 36757867

[3] Akiyama T, Osano K, Mizu-Uchi H, Nakamura N, Okazaki K, Nakayama H, et al. Distal tibial tuberosity arc osteotomy in open-wedge proximal tibial osteotomy to prevent patella infra. Arthrosc Tech. 2019;8:e655–62. 10.1016/j.eats.2019.02.011. PMID: 31334025; PMCID: PMC6624185. PMC6624185 31334025

[4] Otakara E, Nakagawa S, Arai Y, Inoue H, Kan H, Nakayama Y, et al. Large deformity correction in medial open-wedge high tibial osteotomy may cause degeneration of patellofemoral cartilage: A retrospective study. Med (Baltim). 2019;98:e14299. PMID: 30702601; PMCID: PMC6380813. 10.1097/MD.0000000000014299 PMC6380813 30702601

[5] Nakagawa S, Kan H, Arai Y, Komaki S, Hino M, Inoue A, et al. Effect of open-wedge high tibial osteotomy and lateral retinacular release on the articular cartilage of the patellofemoral joint: Analysis using magnetic resonance imaging T2 mapping. J Clin Med. 2025;14:595. 10.3390/jcm14020595. PMID: 39860601; PMCID: PMC11766158. PMC11766158 39860601

[6] Nakamura R, Akiyama T, Takeuchi R, Nakayama H, Kondo E. Medial closed wedge distal femoral osteotomy using a novel plate with an optimal compression system. Arthrosc Tech. 2021;10:e1497–504. 10.1016/j.eats.2021.02.016. PMID: 34258196; PMCID: PMC8252853. PMC8252853 34258196

[7] Nakayama H, Kanto R, Onishi S, Amai K, Ukon R, Tachibana T, et al. Preoperative Ahlbäck radiographic classification grade significantly influences clinical outcomes of double level osteotomy for osteoarthritic knees with severe varus deformity. J Exp Orthop. 2023;10:5. 10.1186/s40634-023-00573-4. PMID: 36695905; PMCID: PMC9877267. PMC9877267 36695905

[8] Kim JH, Lee DK, Park YB. Computed tomography detects hinge fractures after medial opening wedge high tibial osteotomy: A systematic review. Arthroscopy. 2021;37:1337–52. 10.1016/j.arthro.2020.11.031. Epub 2020 Nov 23. PMID: 33242632. 33242632

[9] Fujii Y, Nakagawa S, Arai Y, Inoue A, Kaihara K, Takahashi K. Analysis of the relationship between the morphology of the proximal tibiofibular joint and lateral hinge fracture in open wedge high tibial osteotomy. Knee. 2022;39:10–7. Epub. PMID: 36115178. 10.1016/j.knee.2022.08.003 36115178

[10] Miltenberg B, Puzzitiello RN, Ruelos VCB, Masood R, Pagani NR, Moverman MA, et al. Incidence of complications and revision surgery after high tibial osteotomy: A systematic review. Am J Sports Med. 2024;52:258–68. Epub 2023 Feb 13. PMID: 36779579. 10.1177/03635465221142868 36779579

[11] Anagnostakos K, Mosser P, Kohn D. Infections after high tibial osteotomy. Knee Surg Sports Traumatol Arthrosc. 2013;21:161-9. 10.1007/s00167-012-2084-5. Epub 2012 Jun 23. PMID: 22729805. 22729805

[12] Arai Y, Nakagawa S, Inoue A, Fujii Y, Cha R, Nakamura K, et al. Long-term outcomes and prognostic factors of medial open wedge high tibial osteotomy for medial compartment knee osteoarthritis or osteonecrosis. J Clin Med. 2025;14:2294. 10.3390/jcm14072294. PMID: 40217740; PMCID: PMC11989383. PMC11989383 40217740

[13] Dugdale TW, Noyes FR, Styer D. Preoperative planning for high tibial osteotomy. The effect of lateral tibiofemoral separation and tibiofemoral length. Clin Orthop Relat Res. 1992;274:248–64. PMID: 1729010. 1729010

[14] Kuriyama S, Watanabe M, Nakamura S, Nishitani K, Sekiguchi K, Tanaka Y, et al. Classical target coronal alignment in high tibial osteotomy demonstrates validity in terms of knee kinematics and kinetics in a computer model. Knee Surg Sports Traumatol Arthrosc. 2020;28:1568–78. 10.1007/s00167-019-05575-3. Epub 2019 Jun 21. PMID: 31227865. 31227865

[15] Takeuchi R, Ishikawa H, Miyasaka Y, Sasaki Y, Kuniya T, Tsukahara S. A novel closed-wedge high tibial osteotomy procedure to treat osteoarthritis of the knee: Hybrid technique and rehabilitation measures. Arthrosc Tech. 2014;3:e431–7. 10.1016/j.eats.2014.04.004. PMID: 25264504; PMCID: PMC4175169. PMC4175169 25264504

[16] Chiba K, Yonekura A, Miyamoto T, Osaki M, Chiba G. Tibial condylar valgus osteotomy (TCVO) for osteoarthritis of the knee: 5-year clinical and radiological results. Arch Orthop Trauma Surg. 2017;137:303–10. 10.1007/s00402-016-2609-3. Epub. PMID: 28132085; PMCID: PMC5310550. PMC5310550 28132085

[17] Heinze G, Schemper M. A solution to the problem of separation in logistic regression. Stat Med. 2002;21(16):2409-19. 10.1002/sim.1047. PMID: 12210625. 12210625

[18] Puhr R, Heinze G, Nold M, Lusa L, Geroldinger A. Firth’s logistic regression with rare events: accurate effect estimates and predictions? Stat Med. 2017;36(14):2302–17. 10.1002/sim.7273. Epub 2017 Mar 12. PMID: 28295456. 28295456

[19] van Smeden M, de Groot JA, Moons KG, Collins GS, Altman DG, Eijkemans MJ, Reitsma JB. No rationale for 1 variable per 10 events criterion for binary logistic regression analysis. BMC Med Res Methodol. 2016;16(1):163. 10.1186/s12874-016-0267-3. PMID: 27881078; PMCID: PMC5122171. PMC5122171 27881078

[20] Kawata M, Jo T, Taketomi S, Inui H, Yamagami R, Matsui H et al. Type of bone graft and primary diagnosis were associated with nosocomial surgical site infection after high tibial osteotomy: Analysis of a national database. Knee Surg Sports Traumatol Arthrosc. 2021;29:429 – 36. 10.1007/s00167-020-05943-4. Epub 2020 Apr 1. PMID: 32239271. 32239271

[21] Liu TW, Chiu CH, Chen ACY, Chang SS, Chan YS. Risk factor analysis for infection after medial open wedge high tibial osteotomy. J Clin Med. 2021;10:1727. 10.3390/jcm10081727. PMID: 33923605; PMCID: PMC8073483. PMC8073483 33923605

[22] Berk AN, Gachigi KK, Trofa DP, Piasecki DP, Fleischli JE, Saltzman BM. Early postoperative complications and associated variables after high tibial osteotomy and distal femoral osteotomy: A 15-year experience from a single academic institution. Am J Sports Med. 2023;51:2574–82. 10.1177/03635465231183092. Epub. PMID: 37417330. 37417330

[23] Hancock G, Martin R, Bell L, Broderick J, Dawson M. Infection in osteotomy around the knee: Incidence, management and outcomes in a high-volume case series. Knee Surg Sports Traumatol Arthrosc. 2024;32:1000-7. 10.1002/ksa.12119. Epub. PMID: 38469916. 38469916

[24] Ekhtiari S, Haldane CE, de Sa D, Simunovic N, Musahl V, Ayeni OR. Return to work and sport following high tibial osteotomy: A systematic review. J Bone Joint Surg Am. 2016;98:1568-77. 10.2106/JBJS.16.00036. PMID: 27655985. 27655985

[25] Villatte G, Erivan R, Fournier PL, Pereira B, Galvin M, Descamps S, et al. Opening-wedge high tibial osteotomy with a secure bone allograft (Osteopure™) and locked plate fixation: Retrospective clinical and radiological evaluation of 69 knees after 7.5years follow-up. Orthop Traumatol Surg Res. 2015;101:953–7. 10.1016/j.otsr.2015.09.023. Epub. PMID: 26596418. 26596418

[26] Darees M, Putman S, Brosset T, Roumazeille T, Pasquier G, Migaud H. Opening-wedge high tibial osteotomy performed with locking plate fixation (TomoFix) and early weight-bearing but without filling the defect. A concise follow-up note of 48 cases at 10 years’ follow-up. Orthop Traumatol Surg Res. 2018;104:477–80. Epub. PMID: 29572182. 10.1016/j.otsr.2017.12.021 29572182

[27] Schenke M, Dickschas J, Simon M, Strecker W. Corrective osteotomies of the lower limb show a low intra- and perioperative complication rate-an analysis of 1003 patients. Knee Surg Sports Traumatol Arthrosc. 2018;26:1867-72. 10.1007/s00167-017-4566-y. Epub 2017 May 10. PMID: 28493074. 28493074

[28] Han SB, In Y, Oh KJ, Song KY, Yun ST, Jang KM. Complications associated with medial opening-wedge high tibial osteotomy using a locking plate: A multicenter study. J Arthroplast. 2019;34:439–45. Epub 2018 Nov 14. PMID: 30503322. 10.1016/j.arth.2018.11.009 30503322

[29] Sidhu R, Moatshe G, Firth A, Litchfield R, Getgood A. Low rates of serious complications but high rates of hardware removal after high tibial osteotomy with Tomofix locking plate. Knee Surg Sports Traumatol Arthrosc. 2021;29:3361–7. 10.1007/s00167-020-06199-8. Epub 2020 Aug 12. PMID: 32789527. 32789527

[30] Kinnard MJ, Cohen JS, Quan T, Foran JRH, Sheth NP. Liver disease increases the risk of postoperative complications in patients undergoing aseptic revision total hip and knee arthroplasty. Arthroplast Today. 2024;29:101516. 10.1016/j.artd.2024.101516. PMID: 39363937; PMCID: PMC11447298. PMC11447298 39363937

[31] Kuo SJ, Huang PH, Chang CC, Kuo FC, Wu CT, Hsu HC, et al. Hepatitis B virus infection is a risk factor for periprosthetic joint infection among males after total knee arthroplasty: A Taiwanese nationwide population-based study. Med (Baltim). 2016;95:e3806. 10.1097/MD.0000000000003806. PMID: 27258517; PMCID: PMC4900725. PMC4900725 27258517

[32] Weinstein EJ, Stephens-Shields AJ, Newcomb CW, Silibovsky R, Nelson CL, O’Donnell JA, et al. Incidence, microbiological studies, and factors associated with prosthetic joint infection after total knee arthroplasty. JAMA Netw Open. 2023;6:e2340457. 10.1001/jamanetworkopen.2023.40457. PMID: 37906194; PMCID: PMC10618849. PMC10618849 37906194

[33] Krissheeven M, Nunkoo S, Ramanah M, Robinson J, Banerjee I. Assessing the clinical efficacy of dupilumab and its impact on the quality of life of adult atopic dermatitis patients: A systematic review. Cureus. 2025;17:e79762. 10.7759/cureus.79762. PMID: 40161158; PMCID: PMC11954541. PMC11954541 40161158

[34] Kawata M, Sasabuchi Y, Taketomi S, Inui H, Matsui H, Fushimi K, et al. Atopic dermatitis is a novel demographic risk factor for surgical site infection after anterior cruciate ligament reconstruction. Knee Surg Sports Traumatol Arthrosc. 2018;26:3699–705. 10.1007/s00167-018-4958-7. Epub. PMID: 29725748. 29725748

[35] Durst C, Rajaee L, Chang K, Lee A, Rajaee S. Psoriasis is a risk factor for surgical site infection after primary TKA. Clin Orthop Relat Res. 2024;482:1196–200. Epub. PMID: 38376244; PMCID: PMC11219136. 10.1097/CORR.0000000000003011 PMC11219136 38376244

[36] Xu Z, Li L, Xie P, Gu J, Kang K, Zheng X. Clinical use of tranexamic acid in high tibial osteotomy: A systematic review and meta-analysis. Orthop J Sports Med. 2024;12:23259671241231761. PMID: 38455151; PMCID: PMC10919141. 10.1177/23259671241231761 PMC10919141 38455151

